# Drug-Drug Interaction Extraction via Convolutional Neural Networks

**DOI:** 10.1155/2016/6918381

**Published:** 2016-01-31

**Authors:** Shengyu Liu, Buzhou Tang, Qingcai Chen, Xiaolong Wang

**Affiliations:** Key Laboratory of Network Oriented Intelligent Computation, Harbin Institute of Technology Shenzhen Graduate School, Shenzhen 518055, China

## Abstract

Drug-drug interaction (DDI) extraction as a typical relation extraction task in natural language processing (NLP) has always attracted great attention. Most state-of-the-art DDI extraction systems are based on support vector machines (SVM) with a large number of manually defined features. Recently, convolutional neural networks (CNN), a robust machine learning method which almost does not need manually defined features, has exhibited great potential for many NLP tasks. It is worth employing CNN for DDI extraction, which has never been investigated. We proposed a CNN-based method for DDI extraction. Experiments conducted on the 2013 DDIExtraction challenge corpus demonstrate that CNN is a good choice for DDI extraction. The CNN-based DDI extraction method achieves an *F*-score of 69.75%, which outperforms the existing best performing method by 2.75%.

## 1. Introduction

Drug-drug interactions (DDIs) occur when two or more drugs are taken in combination that alters the way one or more drugs act in human body and may result in unexpected side effects. The unexpected side effects caused by DDIs are always very dangerous (may lead to deaths) and greatly increase healthcare costs. The more DDIs healthcare professionals know, the less medical accidents occur. Therefore, DDIs have always been attracting much attention in drug safety and healthcare management [1]. There are several publicly available databases supporting healthcare professionals to find DDIs. For example, DrugBank [2], which is an online drug database, consists of 8311 drugs entries. Each drug entry contains more than 200 fields, including a DDI field. However, the databases have a few limitations. Firstly, most DDI databases are dictionaries with a DDI field described in text such as DrugBank. The DDIs in these databases cannot be directly accessed like relational databases by healthcare professionals. Secondly, new DDIs are often detected by healthcare professionals and presented in literature, including scientific articles, books, and technical reports [3]. It is impossible for healthcare professionals to find DDIs from the overwhelming amount of literature manually and to keep up-to-date with the latest DDI findings. Therefore, DDI extraction, which detects DDIs in unstructured text and classifies them into predefined categories automatically, has become an increasing interest in medical text mining.

DDI extraction is a typical relation extraction task in natural language processing (NLP). Many methods have been proposed for DDI extraction and can be divided into two categories: rule-based [4] and machine learning-based methods [5, 6]. Rule-based methods use manually defined rules to extract DDIs, whereas machine learning-based methods treat DDI extraction as a standard supervised learning problem over annotated corpora. Compared with rule-based methods, machine learning-based methods usually show better performance and better portability [7]. Due to lack of annotated corpora, early DDI extraction methods are almost all rule-based. For example, Segura-Bedmar et al. [4] defined a set of domain-specific rules to extract DDIs in DrugBank. With the organization of DDIExtraction challenges in 2011 and 2013 [8, 9], machine learning-based methods have been proposed for DDI extraction on the public corpora of the challenges. Both DDIExtraction 2011 and DDIExtraction 2013 are designed to extract drug-drug interactions from biomedical texts. DDIs without type information are labeled in the DDIExtraction 2011 corpus, while, in the DDIExtraction 2013 challenge, DDIs are divided into four types, that is, “*mechanism,*” “*effect,*” “*advice,*” and “*int.*” The top performing systems on these corpora are based on support vector machines (SVM) with a large number of manually defined features [10–12]. For example, the best system of the 2013 DDIExtraction challenge [11] is based on SVM with a hybrid kernel using trigger words, dependency tree and parse tree features, and so forth. The subsequential best system [12] is based on linear SVM with rich features, including word, word pair, dependency graph, parse tree, and noun phrase-constrained coordination features. These systems have to suffer from fussy feature engineering. Most of features used in these systems are usually generated by existing NLP toolkits which are imperfect. Errors caused by the NLP toolkits inevitably propagate in the DDI extraction systems.

Convolutional neural networks (CNN), a robust machine learning method proposed recently which almost does not need manually defined features, has exhibited great potential for many NLP tasks such as sentiment analysis [19], semantic parsing [20], and search query retrieval [21]. However, it has never been used for DDI extraction. In this paper, we deploy CNN for this task. Inputs of the CNN-based method are sentences in which drugs are annotated. The CNN-based method consists of four layers: a look-up table layer, a convolutional layer, a max pooling layer, and a softmax layer. Given a sentence with two drugs of interest, in the look-up table layer, each word is represented by word embeddings [22] and position embeddings [23], and then the sentence is represented by a matrix that concatenates word embeddings and position embeddings of its words in the order of their occurrence. In the convolutional layer, the matrix of the sentence is convolved with filters of different sizes, generating a group of feature vectors. The number of feature vectors is equal to that of filters and the size of each vector is determined by the context window considered. In the max pooling layer, the group of vectors is converted into a new vector by reducing each vector in the group into a feature. Finally, the vector obtained in the max pooling layer is fed to the fully connected softmax layer for classification. The word embeddings used in the look-up table layer are initialized by the “Order” algorithm [22] on the MEDLINE abstracts in 2013 [24], whereas the position embeddings are randomly initialized.

Evaluation on the 2013 DDIExtraction challenge corpus demonstrates that the CNN-based DDI extraction system achieves a precision, a recall, and an *F*-score of 75.72%, 64.66%, and 69.75%, respectively. It outperforms the best performing system by 2.75% in *F*-score, indicating that CNN is a good choice for DDI extraction.

## 2. Methods

**Figure 1:**
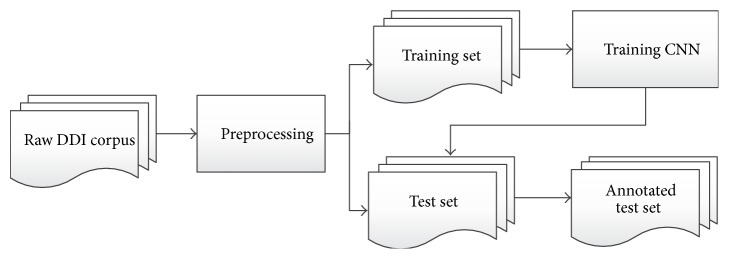
Overall workflow of the CNN-based method for DDI extraction.

DDI extraction is recognized as a multiclass classification problem for all possible interacting pairs of drugs in the same sentence. Each pair of drugs is classified into one of the predefined types of DDIs or classified as a noninteracting pair. Given a sentence with *n* drugs, a total of *C*_*n*,2_ = *n*(*n* − 1)/2 DDI candidates need to be classified.  Figure 1 illustrates the overall workflow of our CNN-based method for DDI extraction.

The preprocessing module first blinds drugs, tokenizes sentences, normalizes tokens, and filters out noninteracting pairs from DDI candidates. Then the CNN module is used for DDI extraction. In the training phase, DDI candidates that annotated in the training set are positive samples with different types, and the other candidates are negative samples. The task of training is to obtain a CNN model on these samples. In the test phase, all DDI candidates are classified into different types of DDIs or non-DDI.

### 2.1. Preprocessing

To ensure generalization of machine learning-based methods, we follow previous studies [12, 25] to blind drugs in a sentence in the following way: the two drugs of interest are replaced by “drug1” and “drug2” in the order of their occurrence, respectively, and all the other drugs are replaced by “drug0.” For example, given a sentence with four drugs, “When *ALFENTA* is administered in combination with other *CNS depressants* such as *barbiturates,* or *tranquilizers,*” where drugs are highlighted in italic, *C*_4,2_ = 6 DDI candidates with context (called instances) are generated, as shown in Table 1.

After drug blinding, we use the Natural Language Toolkit (NLTK) [26] to tokenize sentences and convert all words to lowercase.

Among all DDI instances, there are a large number of negative instances (noninteracting drug pairs with context), which usually affect the performances of machine learning-based DDI extraction systems because of data imbalance problem [27, 28]. Therefore, filtering out negative instances as many as possible is very important for subsequent DDI extraction module. In this study, we define the following four criteria for negative instance filtering. An instance, denoted by “drug1, drug2,” is a negative instance if

1. the two drugs have the same name,
2. one drug is an abbreviation or acronym of the other,
3. the two drugs appear in the same coordinate structure that has more than two drugs as elements,
4. one drug is a special case of the other.

Exact string matching is used to determine whether the first criterion is satisfied, and some simple rules are defined to determine whether any one of the other three criteria is satisfied such as “drug1 (drug2),” “drug1, drug2, and drug0,” and “drug1 such as drug2.” For example, the fourth and fifth instances in Table 1 are negative instances because of criterion 4.

### 2.2. Convolutional Neural Networks for Drug-Drug Interaction Extraction

**Figure 2:**
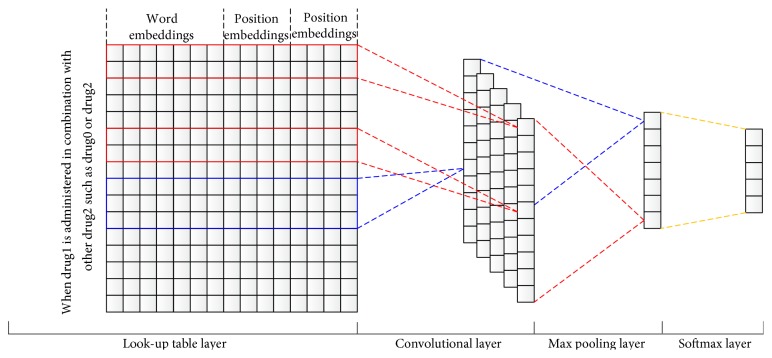
Architecture of the CNN model for DDI extraction.

The CNN model proposed for DDI extraction in this study is a four-layer model (shown in Figure 2), which is a variant of the model for sentence classification in [19]. Besides word embeddings, position embeddings [23] are also integrated into the CNN model in [19] to encode relative distances between words and the two drugs of interest.

#### 2.2.1. Look-Up Table

The CNN model takes DDI instances as input and generates their representation in look-up table layer. As required by CNN, we set all instances to be of the same length by appending padding, denoted by “#,” to short instances. The maximal length of all instances is a proper choice of the same length, denoted by *n*. Given a DDI instance *S* = *w*_1_ *w*_2_ *w*_3_ ⋯ *w*_*n*_ with two drugs of interest (“drug1” and “drug2”) at positions *p*_1_ and *p*_2_, a word *w*_*i*_ is represented by *d*_*w*_-dimensional word embeddings **e**_*w*_*i*__ and 2*d*_*p*_-dimensional position embeddings [**e**_*d*_*i*1__^T^, **e**_*d*_*i*2__^T^]^T^ looked up from corresponding dictionaries. **C** ∈ *ℝ* ^*d*_*w*_×|*V*|^, **D**_1_ ∈ *ℝ* ^*d*_*p*_×(2*n*−1)^, and **D**_2_ ∈ *ℝ* ^*d*_*p*_×(2*n*−1)^, where *V* is the vocabulary and *d*_*i*1_ = *i* − *p*_1_ and *d*_*i*2_ = *i* − *p*_2_ (ranging from −*n* + 1 to *n* − 1) are, respectively, the relative distance between the word and the first drug and that between the word and the second drug. That is, *w*_*i*_ is represented by **x**_*i*_ = [**e**_*w*_*i*__^T^, **e**_*d*_*i*1__^T^, **e**_*d*_*i*2__^T^]^T^. Then the instance is represented by a matrix that concatenates the word embeddings and position embeddings of its words in the order of their occurrence, denoted by **x** = [**x**_1_, **x**_2_, **x**_3_,…, **x**_*n*_] of size (*d*_*w*_ + 2*d*_*p*_) × *n*. For the two types of embeddings, word embeddings can be initialized by employing unsupervised word embeddings algorithm on large-scale unannotated texts, whereas position embeddings only can be randomly initialized.

#### 2.2.2. Convolution

The matrix of a DDI instance (i.e., **x**) is fed to the convolutional layer to generate features by convolving **x** with filters of different sizes. Given a filter of size *k*, **t** ∈ *ℝ* ^(*d*_*w*_+2*d*_*p*_)×*k*^, for example, the following feature *f*_*i*_ can be generated by applying convolution operator to a context window of *k* words:

(1) $$\begin{array}{c} f_{i}=\tanh\left(\;t\cdot x_{i:i+k-1}+b\;\right), \end{array}$$

where **x**_*i*:*i*+*k*−1_ denotes the matrix [**x**_*i*_, **x**_*i*+1_, **x**_*i*+2_,…, **x**_*i*+*k*−1_] (representation of words in the context window), *b* ∈ *ℝ* is a bias, and tanh is the hyperbolic tangent function. When filter **t** is applied to all possible context windows of *k* words (i.e., *i* ranging from 1 to *n* − *k* + 1), a feature vector **f** = [*f*_1_, *f*_2_, *f*_3_,…, *f*_*n*−*k*+1_] (**f** ∈ *ℝ* ^*n*−*k*+1^) is generated. As there are various types of filters of different sizes, we can obtain a group of feature vectors. The number of feature vectors is equal to the number of filters.

#### 2.2.3. Max Pooling

The max pooling layer extracts the most important feature from each feature vector to reduce the computational complexity of subsequent layers. Concretely, the feature of maximum value $\hat{f}=\max\left\{f_{1},f_{2},f_{3},\ldots ,f_{n-k+1}\right\}$ is extracted to represent a feature vector **f** = [*f*_1_, *f*_2_, *f*_3_,…, *f*_*n*−*k*+1_]. Correspondingly, if there are *l* feature filters, the matrix of a DDI instance (i.e., **x**) is converted into a new vector of length *l*, denoted by $z=[\hat{f_{1}},\hat{f_{2}},\hat{f_{3}},\ldots ,\hat{f_{l}}]$, where $\hat{f_{i}}$ is the feature extracted from the *i*th feature vector.

#### 2.2.4. Softmax Regression

To prevent neural networks from overfitting, we follow [19] to randomly drop out units (along with their connections) from the networks during training. The feature vector **z** obtained by max pooling is not directly fed to the fully connected softmax layer for classification. Firstly, we randomly set each element of **z** to zero with a probability *p* (following the Bernoulli distribution) and obtain a new feature vector **z**_*d*_. Then the vector **z**_*d*_ is fed to the fully connected softmax layer. At test time, the feature vector **z** is directly fed to the softmax layer for classification without dropout.

#### 2.2.5. Model Training

The following parameters of the CNN model need to be updated during training: the word embeddings matrix, the position embeddings matrixes, the filters, and the weight matrix of the softmax layer. We use stochastic gradient descent with shuffled minibatches and the AdaDelta update rule as [19] to learn the parameters. At each gradient descent step, we rescale the weight vectors of the softmax layer when their *l*_2_-norms exceed a certain threshold.

## 3. Experiments

### 3.1. Dataset

The CNN-based DDI extraction system is developed and evaluated on the DDI corpus of the 2013 DDIExtraction challenge [29], which is composed of 730 DrugBank documents and 175 MEDLINE abstracts about DDIs. The corpus is split into two parts: a training set (572 DrugBank documents and 142 MEDLINE abstracts) for system development and a test set (158 DrugBank documents and 33 MEDLINE abstracts) for system evaluation (see Table 2). All drugs and pairs of drugs in each sentence are annotated. Among the pairs of drugs (totally 33508), 5000 interacting pairs (i.e., DDIs) are classified into the following four types: mechanism, effect, advice, and int. The definitions of the four types of DDIs are as follows.

*(i) Mechanism*. Mechanism is assigned when pharmacokinetic mechanism of a DDI is described.

*(ii) Effect.* Effect is assigned when effect of a DDI is described.

*(iii) Advice.* Advice is assigned when a recommendation or advice regarding a DDI is given.

*(iv) Int.* Int is assigned when the sentence simply states that a DDI occurs and does not provide any information about the DDI.

### 3.2. Experimental Settings

As four types of DDIs are defined in the corpus of the 2013 DDIExtraction challenge, DDI instances need to be classified into five categories: mechanism, effect, advice, int, and noninteracting, corresponding to the output of the last layer of the CNN model.

The word embeddings matrix used in our experiments is initialized by an unsupervised word embeddings learning algorithm “Order” [22] on 17.3-gigabyte unannotated article abstracts extracted from MEDLINE released in 2013 [24]. We also adopt the NLTK to preprocess the abstracts, including splitting them into sentences, tokenizing the sentences, and converting all words to lowercase. Finally, we obtain 110 million sentences with 2.8 billion words from a vocabulary of size 1.99 million. Following previous works [19], we set the dimension of word embeddings to 300 and randomly initialized word embeddings of words not present in the vocabulary. For the position embeddings matrixes, we follow [23] to randomly initialize the position embeddings and determine the dimension of position embeddings heuristically (finally set to 10).

The maximal length of the DDI instances is set to 150, that is, the maximal length of sentences in the DDIExtraction 2013 corpus. Following [19], we used three kinds of filters for convolution; that is, *k* is set to 3, 4, and 5 for filter **t** ∈ *ℝ* ^(*d*_*w*_+2*d*_*p*_)×*k*^, and we used 200 filters of each kind at the convolutional layer. The dropout rate (*p*), *l*_2_-norm threshold, and minibatch size are, respectively, set to 0.5, 3, and 50, the same as [19]. Our CNN-based DDI extraction system will be released after the publication of this study.

To investigate the effect of different factors, we start with a baseline system without using position embeddings and negative instance filtering module and then add them gradually. We also compare our system with other state-of-the-art systems. The performances of all DDI extraction systems are measured by precision (*P*), recall (*R*), and *F*-score (*F*), which are calculated by the evaluation tool provided by the 2013 DDIExtraction challenge organizers [30].

### 3.3. Experimental Results

The overall precision, recall, and *F*-score of our system are 75.72%, 64.66%, and 69.75%, as shown in Table 3, where the best performances are emphasized in bold. On the DrugBank subset, our system achieves an *F*-score of 71.52%, which is higher than that on the MEDLINE subset by 19.40%. Among four types of DDIs, our system performs best on advice instances and worst on int instances. The difference between the *F*-scores on these two types of DDIs achieves 31.37% (77.75% versus 46.38%).

Both position embeddings and negative instance filtering improve the overall performance of the CNN-based DDI extraction system. The improvements from them are 2.01% (67.01% versus 65.00%) and 0.62% (65.62% versus 65.00%) in *F*-score, respectively. When both of them are added to the baseline system, the CNN-based system is further improved by a total increase of *F*-score of 4.75% (69.75% versus 65.00%). The system using both position embeddings and negative instance filtering shows much better performance than other systems on the DrugBank subset but worse performance than the system only using negative instance filtering by 3.05% on the MEDLINE subset. On all the four types of DDIs except int, the system using both position embeddings and negative instance filtering achieves better *F*-score than other systems. On int, the baseline system achieves best performance.

Compared with other state-of-the-art systems, including the best existing system and all participating systems of the 2013 DDIExtraction challenge (8 systems), our CNN-based system shows much better performance. It outperforms the current best system (Kim et al. [12]) by 2.75% and the best system of the 2013 DDIExtraction challenge (FBK-irst [11]) by 4.65% in *F*-score (see Table 4), mainly due to much higher precision. Top performing systems in Table 4 (e.g., Kim et al. [12], FBK-irst [11], and WBI [13]) are all based on SVM with a large number of manually defined features such as word, word pair, and dependency graph, as mentioned in Section 1. 

## 4. Discussion

In this study, we propose a CNN-based system to extract DDIs in biomedical texts. To the best of our knowledge, it is the first time to use CNN for DDI extraction. As shown in Table 4, our CNN-based system outperforms all existing systems, most of which are based on SVM with various features such as syntactic feature [11, 12] and features derived from external lexical resources [13, 14]. Compared with the state-of-the-art SVM-based systems, the advantage of our CNN-based system lies in that it does not use any manually defined features generated by existing NLP toolkits. The features used in the CNN-based system (i.e., word embeddings and position embeddings automatically learnt during training) may contain other useful information beyond the manually defined features. Moreover, they effectively avoid errors caused by existing NLP toolkits.

Position embedding improves the performance of our system on the DrugBank subset, but not on the MEDLINE subset. The main reason is that the position distribution of words in the DrugBank subset is more similar to that in the training set than the MEDLINE subset. To prove this point, we compare the average distance between two drugs of interest in the training set with that in the two test subsets and find that the difference between the average distance in the training set (18.06) and the average distance in the DrugBank subset (15.07) is much smaller than that between the training set and the MEDLINE subset (8.55).

The same as previous studies [11, 27], negative instance filtering is beneficial to our system. The negative instance filtering module used in our system removes a large number of negative instances, but almost no positive instances. In the training set, 11206 out of 23771 negative instances are correctly filtered out. In the test set, 2698 out of 4737 negative instances are correctly filtered out, whereas only 7 out of 979 positive instances are wrongly filtered out. On the whole, more than 50% negative instances are correctly filtered out, but less than 0.2% positive instances are wrongly filtered out.

Our system shows much better performance on the DrugBank subset compared to the MEDLINE subset. There may be two reasons: (1) MEDLINE abstracts are usually written in scientific language. Long and complex sentences are commonly used in MEDLINE abstracts. In contrast, sentences in DrugBank are usually short and concise; (2) samples in the training set from MEDLINE are much less than DrugBank.

It is easy to understand that our system performs worst on int instances because of their proportionally small number among four types of DDI instances. The int instances only account for 4.7% (189/4021) in the training set. A possible direction for improvement is to take the imbalanced distributions of different types of instances into account like [31].

Although our system outperforms all other existing systems, there also are a large number of errors in our system (listed in Table 5, where the numbers on the two sides of plus signs are negative instances predicted by the CNN model and negative instance filtering module, resp.). Most of errors occur between positive instances and negative instances. 277 out of 979 positive instances are wrongly classified into negative instances (false negative instances). 134 negative instances are wrongly classified into positive instances (false positive instances). A small number of errors between four different types of DDIs (69 out of 979) occur in our system. Among these errors, 39 int instances are wrongly classified into effect instances, accounting for 56.52%. Reducing errors between positive instances and negative instances will greatly improve the CNN-based DDI extraction system, which is part of our future work.

## 5. Conclusions

In this paper, we propose a CNN-based method for DDI extraction. Word embeddings and position embeddings, which capture the semantic information of words and relative distances between words and two drugs of interest, respectively, are used to represent DDI instances. Experiments on the 2013 DDIExtraction challenge corpus demonstrate that the proposed CNN-based method outperforms other state-of-the-art methods on DDI extraction. It is the first time to apply CNN to DDI extraction. In our CNN-based method, not only word embeddings but also position embeddings are considered. Both of them do not rely on any existing NLP toolkits.

## Supplementary Material

Table S1 compares the proposed method and other leading methods by instance type. It can be seen that the proposed method outperforms other methods for extracting DDI instances of the “Mechanism”, “Effect” and “Advice” type.

## Figures and Tables

**Table 1:** DDI candidates in a sentence after drug blinding.

Drug pair	DDI candidate with context after drug blinding (i.e., instance)
(ALFENTA, CNS depressants)	When *drug1* is administered in combination with other *drug2* such as drug0 or drug0
(ALFENTA, barbiturates)	When *drug1* is administered in combination with other drug0 such as *drug2* or drug0
(ALFENTA, tranquilizers)	When *drug1* is administered in combination with other drug0 such as drug0 or *drug2*
(CNS depressants, barbiturates)	When drug0 is administered in combination with other *drug1* such as *drug2* or drug0
(CNS depressants, tranquilizers)	When drug0 is administered in combination with other *drug1* such as drug0 or *drug2*
(barbiturates, tranquilizers)	When drug0 is administered in combination with other drug0 such as *drug1* or *drug2*

**Table 2:** Statistics of the DDI corpus of the 2013 DDIExtraction challenge.

	Training set		Test set	
	DrugBank	MEDLINE	DrugBank	MEDLINE
Documents	572	142	158	33
Pairs	26005	1787	5265	451
Positive DDIs	3789	232	884	95
Negative DDIs	22216	1555	4381	356
Mechanism	1257	62	278	24
Effect	1535	152	298	62
Advice	818	8	214	7
Int	179	10	94	2

**Table 3:** Performance of the CNN-based DDI extraction systems (%).

Systems	Baseline			Baseline + position embeddings			Baseline + negative instance filtering			Our system		
	*P*	*R*	*F*	*P*	*R*	*F*	*P*	*R*	*F*	*P*	*R*	*F*
Mechanism	**81.22**	52.98	64.13	79.65	59.60	68.18	71.76	62.25	66.67	79.50	**62.91**	**70.24**
Effect	65.38	61.39	63.32	67.44	64.44	65.91	63.10	65.56	64.31	**68.66**	**70.00**	**69.33**
Advice	**86.90**	66.06	75.06	84.97	66.52	74.62	79.06	68.33	73.30	84.57	**71.95**	**77.75**
Int	**84.21**	**33.33**	**47.76**	76.19	33.33	46.38	72.73	33.33	45.71	76.19	33.33	46.38
DrugBank	76.13	59.16	66.58	76.70	62.56	68.91	70.58	63.24	66.71	**77.02**	**66.74**	**71.52**
MEDLINE	**66.67**	37.89	48.32	59.38	40.00	47.80	60.76	**50.53**	**55.17**	61.43	45.26	52.12
Overall	75.44	57.10	65.00	75.29	60.37	67.01	69.69	62.00	65.62	**75.72**	**64.66**	**69.75**

**Table 4:** Comparison between our CNN-based system and other state-of-the-art systems (%).

Systems	DrugBank			MEDLINE			Overall		
	*P*	*R*	*F*	*P*	*R*	*F*	*P*	*R*	*F*
Our system	**77.02**	66.74	**71.52**	**61.43**	**45.26**	**52.12**	**75.72**	64.66	**69.75**
Kim et al. [12]	—	—	69.80	—	—	38.20	—	—	67.00
FBK-irst [11]	66.70	**68.60**	67.60	41.90	37.90	39.80	64.60	**65.60**	65.10
WBI [13]	65.70	60.90	63.20	45.30	30.50	36.50	64.20	57.90	60.90
UTurku [14]	73.80	53.50	62.00	59.30	16.80	26.20	73.20	49.90	59.40
NIL_UCM [15]	56.60	57.90	57.30	35.70	15.80	21.90	55.70	53.80	54.80
UC3M	51.80	59.80	55.50	26.50	28.40	27.40	49.50	56.80	52.90
UWM-TRIADS [16]	45.20	52.40	48.50	31.20	32.60	31.90	43.90	50.50	47.00
SCAI [17]	54.60	40.40	46.40	62.50	31.60	42.00	55.10	39.50	46.00
UColorado_SOM [18]	28.80	44.10	34.90	17.30	41.10	24.40	27.20	43.80	33.60

**Table 5:** Prediction Results of our CNN-based DDI extraction system.

Gold standard annotation		Prediction results				
Type	Total number	Mechanism	Effect	Advice	Int	Negative
Mechanism	302	190	8	7	0	96 + 1
Effect	360	6	252	3	1	94 + 4
Advice	221	2	1	159	2	55 + 2
Int	96	0	39	0	32	25
Negative	4737	41	67	19	7	1905 + 2698
