# Supplementary material for: Drug-Drug Interaction Extraction via Convolutional Neural Networks
Source: Comput Math Methods Med. 2016 Jan 31;2016:6918381. doi: 10.1155/2016/6918381 (PMC4752975; doi:10.1155/2016/6918381)
Supplement: Supplementary file 1 — Table S1 compares the proposed method and other leading methods by instance type. It can be seen that the proposed method outperforms other methods for extracting DDI instances of the “Mechanism”, “Effect” and “Advice” type. [file 6918381.f1.pdf]

Table S1. F-scores of our system and the top performing systems for each type of DDIs (%).

	**Our system**	**Kim Sun**	**FBK-irst**	**WBI**
Mechanism	**70.24**	69.30	67.90	61.80
Effect	**69.33**	66.20	62.80	61.00
Advice	**77.75**	72.50	69.20	63.20
Int	46.38	48.30	**54.70**	51.00
